# *VDR* polymorphisms influence immunological response in HIV-1+ individuals undergoing antiretroviral therapy

**DOI:** 10.1590/1678-4685-GMB-2017-0289

**Published:** 2019-06-27

**Authors:** Ronaldo Celerino da Silva, Neyla Maria Pereira Alves, Jorge José de Souza Pereira, Antonio Victor Campos Coelho, Luiz Cláudio Arraes, Lucas André Cavalcanti Brandão, Sergio Crovella, Jaqueline de Azevêdo Silva

**Affiliations:** 1 Laboratorio de Immunopatologyia Keizo Asami (LIKA), Universidade Federal de Pernambuco, Recife, PE, Brazil; 2 Departamento of Patologia, Universidade Federal de Pernambuco, Recife, PE, Brazil; 3 Departamento de Genética, Universidade Federal de Pernambuco, Recife, PE, Brazil; 4 Instituto de Medicina Integral de Pernambuco Professor Fernando Figueira, Recife, PE, Brazil

**Keywords:** VDR, HIV-1, ARVs, SNPs, CD4 recovery

## Abstract

Vitamin D exerts an immuno-modulatory activity on several immune system cells through the vitamin D receptor (VDR). Herein, we verified that age and a therapeutic regimen containing protease inhibitors are associated with failures in antiretroviral therapies (ARVs). In addition, we assessed whether a *VDR* SNP (rs11568820: C allele and CC genotype) and GC (rs2228570-rs11568820) allelic combinations are associated with immunological failure (*p* < 0.05). Our findings suggest a possible role of *VDR* SNPs on immunological failure in HIV-1+ individuals undergoing regular ARVs.

Antiretroviral therapies (ARVs) have changed the landscape of Human Immunodeficiency Virus (HIV) treatment in the last years. ARVs act by boosting immune functions and reducing morbidity and mortality by suppressing viral replication (Palella *et al.*, 2006). There is no *“one fits all*” therapy, since some individuals receiving ARVs are not able to suppress the viral load to undetectable levels as expected (virological failure) (Coelho *et al.*, 2013). On the other hand, some individuals with undetectable viral load, cannot recover the quasi-normal CD4+ T-cell count (immunological failure) (Kelley *et al.*, 2009). These phenomena might be due to the hosts’ genetics, immunological factors, and viral profiles (Geretti *et al.*, 2009; Robbins *et al.*, 2009).

1,25-dihydroxy vitamin D (1,25[OH]_2_D) exerts potent immunologic roles in both innate and adaptive responses. Its binding with vitamin D receptor (VDR) promotes a series of intracellular events culminating in the modulation of expression levels of various target genes. These effects may promote the control of intracellular pathogens, mainly by producing antimicrobial peptides, and stimulating phagocytic activity in macrophages (Prietl *et al.*, 2013).

In HIV-1 infection, variable levels of 1,25[OH]_2_D may decrease the immune system activation during the acute phase, or induce a less effective T-helper lymphocyte response throughout the chronic phase (Appay and Sauce, 2008; Giusti and Penco, 2011). Indeed, individuals with adequate levels of this vitamin, have been seen to better counteract HIV replication (Lake and Adams, 2011).

In addition, ARVs may alter the circulating levels of 25-hydroxy vitamin D (25[OH]D) (1,25[OH]_2_D metabolic precursor), as these compounds share common cytochrome P450 enzymes involved in their synthesis (1,25[OH]_2_D) and excretory (ARVs) metabolic pathways (Mueller *et al.*, 2010; Welz *et al.*, 2010). Interestingly, 1,25[OH]_2_D insufficiency/deficiency has been associated with the usage of ARVs (Cozzolino *et al.*, 2003; Brown and McComsey, 2010).

Single nucleotide polymorphisms (SNPs) in the *VDR* gene (12q13.11) have been associated to susceptibility and progression of HIV/AIDS, since they may alter gene function and compromise the role of 1,25[OH]_2_D (de la Torre *et al.*, 2008; Alagarasu *et al.*, 2009; Aguilar-Jiménez *et al.*, 2013; Laplana *et al.*, 2014). However, there are no reports associating these genetic variants with successful treatment and immune recovery.

In this work, we investigated the distribution of *VDR* SNPs in HIV-1 infected individuals (HIV-1+) to unravel their relation with treatment response and immune recovery.

We analyzed 195 HIV-1+ individuals from Recife and minor towns of the state of Pernambuco (Northeastern Brazil), recruited from 2011 to 2014 at the Institute of Integral Medicine Professor Fernando Figueira (Human Research Ethics Committee register nr. 2629-13). Individuals lacking pre-existing viral hepatitis co-infection, and who have adhered for at least one year to ARVs have been selected for this study (Coelho *et al.*, 2013). The studied population was stratified into 132 individuals with undetectable viral load (< 50 copies/mL) (virological success/suppression) and 63 individuals having detectable viral load (> 50 copies/mL) (virological failure). In addition, based on the comparison between CD4+ T-cell count before treatment (baseline) and after one year of treatment (Li *et al.*, 2011), individuals possessing virological suppression were divided as immunological success (65) (recovery of CD4+ > 200 cells/mm^3^) and immunological failure (67) (non-recovery CD4+ < 200 cells/mm^3^).

Individuals manifesting virological failure were mostly female (66.7%), with a median age of 35 years (*p*=0.009, Mann-Whitney test), and receiving therapeutic regimens containing protease inhibitors (2 non-nucleoside reverse transcriptase inhibitor + protease inhibitor [NNRTI+PI]; 55.6%, *p*=0.037, Chi-square test). Furthermore, the NNRTI-containing regimen resulted more frequently in individuals with immunological failure (68.7%) in comparison to the immunological successful ones (53.1%, *p*=0.047, Chi-square test) (Table 1).

**Table 1:** Clinical and Epidemiological characteristics of the study population.

	Virologic Response			Immunological Response		
Characteristics	Success (n=132)	Failure (n=63)	Univariate analysis	Success (n=65)	Failure (n=67)	Univariate analysis
Sex, n (%)						
Female	72 (54.5)	42 (66.7)	X^2^=2.1052, df=1, *p*-value= 0.147	40 (61.5)	32 (47.8)	X^2^=1.7736, df=1, *p*-value=0.183
Male	60 (45.5)	21 (33.3)		25 (38.5)	35 (52.2)	
Age						
Median years (IQR)	38.0 (34.0-46.0)	35.0 (31.5-41.0)	W=3201, *p*-value=0.009*	37.0 (34.0-46.0)	38.0 (34.0-45.0)	W=2015, *p*-value=0.528
Weight						
Median kilograms (IQR)	62.0 (54.2-74.0)	62.7 (55.0-73.2)	W=4043.5, *p*-value=0.500	63.0 (53.0-74.0)	62.0 (56.0-74.0)	W=1858, *p*-value=0.630
Baseline viral load						
Median log10 copies/mL(IQR)	4.40 (3.02-5.10)	3.78 (2.85-4.91)	W=3417.5, *p*-value=0.526	4.52 (3.68-5.06)	4.31 (2.36-5.11)	W=1741, *p*-value=0.451
CD4+ T cells count baseline						
Median cells/mm^3^ (IQR)	282.0 (165.0-365.0)	291.0 (179.0-410.0)	W=3883, *p*-value=0.769	282 (200.5-433.0)	282.5 (126.0-343.7)	W=1567, *p*-value=0.178
HAART regimens, n (%)						
NNRTI use	80 (61.1)	28 (44.4)	X^2^= 4.3617, df=1, *p*-value=0.037*	32 (53.1)	43 (68.7)	X^2^= 3.9558, df=1, *p*-value=0.047*
PI use	50 (38.9)	35 (55.6)		29 (46.9)	17 (31.3)	

n= Sample size; IQR = Interquartile range; X^2^= Result from chi-squared test; df = Degrees of freedom; W =Result from Mann-Whitney test; * = Significant *p*-value.

Studies in Latin American populations (Bello *et al.*, 2011; Alave *et al.*, 2013; Cesar *et al.*, 2015) also identified as risk factors for virological failure parameters such as younger age (20 years), infection pathway (Cesar *et al.*, 2015), antiretroviral use history before starting a new therapy regimen, therapy change due to toxicity, opportunistic infections, baseline CD4+ T-cells count below 100 cells/μL, adherence, advanced clinical stage (Alave *et al.*, 2013), geographical origin, and *Mycobacterium tuberculosis* co-infection (Bello *et al.*, 2011).

Besides the clinical-epidemiological characteristics, a panel of four tag-SNPs in the intronic region within the *VDR* gene (rs3890733, rs4760648, rs1540339, rs2248098) and two functional ones, namely rs2228570 (Fok1 – missense variation – stat lost) and rs11568820 (Cdx2 – regulatory variation) were studied. SNP selection was based on public data bank as well as literature data (de la Torre *et al.*, 2008; Alagarasu *et al.*, 2009; Laplana *et al.*, 2014; Xu *et al.*, 2015). All variants were genotyped using allele-specific fluorogenic probes by real-time PCR and presented minor allele frequency (MAF) ≥ 10% in Caucasian (CEU) and Yoruba (YRI) populations, considering them as proxies to Brazil’s ancestral populations (Coelho *et al.*, 2015).

Allelic and genotypic frequencies of *VDR* SNPs in treatment and immunological response groups are reported in Table 1. All the considered SNPs studied were in accordance with Hardy-Weinberg equilibrium in all groups, except for SNP rs3890733. No significant differences were observed for the tested SNPs relative to treatment response, but the SNP rs11568820 (C > T) was found to be associated with immune recovery.

The C allele (rs11568820) was significantly more frequent in individuals presenting immunological failure (62.1%), rather than in those showing immunological success (48.4%; OR=1.74; 95%CI=1.01-3.03; *p*=0.037, Fisher’s exact test). Similarly, the C/C genotype was significantly more frequent in immunological failure (34.5%) in comparison to individuals with immunological success (19.7%; OR=3.78; 95%CI=1.03-15.57*; p*=0.045, Fisher’s exact test) (Table 2).

**Table 2:** Allelic and genotypic frequencies of *VDR* SNPs in HIV-1+ patients in HAART treatment as to the virological and immunological response.

SNPs	Virological		Fisher’s Exact Test	Immunological		Fisher’s Exact Test
	Success n (%)	Failure n (%)	OR (95% CI),*p-*value	Success n (%)	Failure n (%)	OR (95% CI),*p-*value
rs2248098						
G	125 (54.3)	52 (46.4)	Reference	62 (55.4)	63 (53.4)	Reference
A	105 (45.7)	60 (53.6)	1.37 (0.85-2.22), 0.205	50 (44.6)	55 (46.6)	1.08 (0.62-1.88), 0.792
GG	34 (29.6)	12 (21.4)	Reference	20 (35.7)	14 (23.7)	Reference
GA	57 (49.6)	28 (50.0)	1.39 (0.59-3.41), 0.551	22 (39.3)	35 (59.3)	2.25 (0.88-5.93), 0.082
AA	24 (20.9)	16 (28.6)	1.87 (0.69-5.22), 0.249	14 (25.0)	10 (16.9)	1.02 (0.31-3.34), 1.000
rs1540339						
C	167 (69.6)	77 (67.5)	Reference	85 (70.8)	82 (68.3)	Reference
T	73 (30.4)	37 (32.5)	1.10 (0.66-1.82), 0.713	35 (29.2)	38 (31.7)	1.12 (0.62-2.03), 0.779
CC	59 (49.2)	28 (49.1)	Reference	31 (51.7)	28 (46.7)	Reference
CT	49 (40.8)	21 (36.8)	0.90 (0.43-1.88), 0.863	23 (38.3)	26 (43.3)	1.25 (0.55-2.86), 0.699
TT	12 (10.0)	8 (14.0)	1.40 (0.44-4.23), 0.601	6 (10.0)	6 (10.0)	1.10 (0.26-4.67), 1.000
rs2228570						
G	180 (69.8)	87 (71.3)	Reference	83 (65.9)	97 (73.5)	Reference
A	78 (30.2)	35 (28.7)	0.93 (0.56-1.52), 0.811	43 (34.1)	35 (26.5)	0.70 (0.39-1.23), 0.222
GG	63 (48.8)	29 (47.5)	Reference	27 (42.9)	36 (37.9)	Reference
GA	54 (41.9)	29 (47.5)	1.16 (0.59-2.30), 0.748	29 (46.0)	25 (37.9)	0.65 (0.29-1.43), 0.269
AA	12 (9.3)	3 (4.9)	0.54 (0.09-2.24), 0.545	7 (11.1)	5 (7.6)	0.54 (0.12-2.22), 0.359
rs4760648						
T	98 (53.3)	40 (58.8)	Reference	52 (55.3)	46 (51.1)	Reference
C	86 (46.7)	28 (41.2)	0.80 (0.43-1.45), 0.477	42 (44.7)	44 (48.9)	1.18 (0.66-2.20), 0.658
TT	26 (28.3)	13 (38.2)	Reference	14 (29.8)	12 (26.7)	Reference
TC	46 (50.0)	14 (41.2)	0.61 (0.23-1.65), 0.356	24 (51.1)	22 (48.9)	1.07 (0.37-3.14), 1.000
CC	20 (21.7)	7 (20.6)	0.70 (0.20-2.33), 0.593	9 (19.1)	11 (24.4)	1.41 (0.38-5.39), 0.767
rs3890733 §						
C	149 (64.8)	67 (67.0)	Reference	78 (67.2)	71 (62.3)	Reference
T	81 (35.2)	33 (33.0)	0.91 (0.53-1.53), 0.801	38 (32.8)	43 (37.7)	1.24 (0.70-2.22), 0.490
CC	58 (50.4)	26 (52.0)	Reference	32 (55.2)	26 (45.6)	Reference
CT	33 (28.7)	15 (30.0)	1.01 (0.43-2.32), 1.000	14 (24.1)	19 (33.3)	1.66 (0.65-4.34), 0.280
TT	24 (20.9)	9 (18.0)	0.84 (0.30-2.19), 0.824	12 (20.7)	12 (21.1)	1.23 (0.42-3.56), 0.808
rs11568820						
T	109 (45.4)	50 (46.3)	Reference	63 (51.6)	44 (37.9)	Reference
C	131 (54.6)	58 (53.7)	0.96 (0.60-1.56), 0.908	59 (48.4)	72 (62.1)	1.74 (1.01-3.03), 0.037*
TT	21 (17.5)	12 (22.2)	Reference	14 (23.0)	6 (10.3)	Reference
CT	67 (55.8)	26 (48.1)	0.68 (0.27-1.75), 0.384	35 (57.4)	32 (55.2)	2.11 (0.66-7.56), 0.203
CC	32 (26.7)	16 (29.6)	0.88 (0.31-2.47), 0.815	12 (19.7)	20 (34.5)	3.78 (1.03-15.57), 0.045*

n = Sample size; OR = Odds ratios; 95% CI = 95% Confidence interval; * = significant *p-*value; § = not Hardy-Weinberg Equilibrium.

No haplotype block was observed for *VDR* functional SNPs in all studied groups (D’ > 0.20). Allelic combination analyses revealed that only the GC allele combination (rs2228570-rs11568820) was associated with immunological failure (OR=2.19; 95%CI=1.09-4.45; *p*=0.023, Fisher’s exact test) (Table S1).

Two multivariate analyses were performed containing variables with *p* ≤ 0.2 obtained during univariate analyses. Age, gender, antiretroviral drugs classes, and rs2248098 genotypes were included as clinic-epidemiological and genetic variables to assess whether they influenced the overall virological response, but no significant associations were found. The second analysis included the pre-treatment (baseline) CD4+ T-cells count, gender, and antiretroviral drugs classes. In the second multivariate analysis we intended to assess whether rs2248098 and rs11568820 genotypes had any influence on immunological response. Again, the rs11568820 C/C genotype was associated with immunological failure (OR=6.00; 95%CI=1.52-23.72; *p-*value=0.01) (Table S2).

None of the studied SNPs were involved in viral load control before treatment and CD4+ T-cell count baseline, even when classified according to virological treatment response or immunological recovery (*p-*values > 0.05; Kruskal-Wallis test, data not shown).

The C allele and C/C genotype of SNP rs11568820 and the GC allelic combination (rs2228570-rs11568820) were associated with immunological failure in individuals that achieved the viral load suppression following ARVs. The understanding of this immunological impairment in viral-suppressed HIV-1 individuals remains elusive mainly due to its multifactorial character. In our work, we addressed a SNP as a candidate for contributing in the immune recovery modulation. The rs11568820 SNP (known as Cdx-2), located within the VDR promoter region, acts at the transcriptional level. Therefore, the presence of the C allele (rs11568820) may result in a decreased transcriptional activity and, consequently, in lower VDR expression (Arai *et al.*, 2001).

Finally, the levels of 25(OH)D in plasma were obtained from medical records of 31 individuals deriving from our sample groups (19 immunological success and 12 immunological failure). A Cochran-Mantel-Haenszel test was applied to assess whether the interaction between 25(OH)D plasma levels and VDR genotypes had an impact on immunological failure risk, thus following the hypothesis that some VDR genotypes would not respond to 25(OH)D levels, even if they are adequate. However, no significant differences were found (success group median=35.2, IQR=28.1-41.1; failure group median=32.9, IQR=31.4-41.0; W=109; *p-*value=0.86, Table S3). Next, we stratified the individuals according to *VDR* genotypes and insufficient/sufficient status and evaluated, through a Cochran-Mantel-Haenszel test, whether the genotypes versus 25(OH)D influenced the risk of immunological failure. Also in this case, no associations were observed. Even deficient 25(OH)D levels have been found not to be associated with immunological failure, irrespective of VDR genotype (data not shown).

We did not identify relationships between plasma levels and immunological failure, in spite of a *VDR-*associated genotype (rs11568820). This fact may be related to the small number of individuals used in the dosage and/or the absence of a proper classification of studied individuals referred to the infection stage, which in our population is almost impossible to perform, since the vast majority of HIV infected cases have a late diagnosis, thus arriving at health services presumably in a chronic stage, and finally the lack of control regarding seasonal variation in vitamin D. In our cohort it is assumed that such inter-individual variation is small, as there is a regular incidence of solar rays in the geographic region.

Nonetheless, we believe that individuals with immunological failure and *VDR* genotype (CC – rs11568820), related to low expression of *VDR*, can impair 1,25[OH]_2_D action, since its activity occurs through VDR binding (Selvaraj *et al.*, 2012). A reduced 1,25[OH]_2_D activity compromises cell-mediated immune response, phagocytic activity in macrophages (Deluca and Cantorna 2001; Prietl *et al.*, 2013), suppresses T-cell activation, downregulates pro-inflammatory cytokine production, and decreases anti-microbial defence by peptides, which in turn impair viral replication (Beard *et al.*, 2011).

Furthermore, Chandel *et al.* (2013) demonstrated that down-regulation of VDR expression in T-cells deriving from HIV-1+ individuals is due to hypermethylation in the promoter region. These epigenetic alterations increase the activation of the renin angiotensin system (RAS) and reactive oxygen species (ROS) production, inducing double-strand breaks (DSBs) and attenuated DNA repair response, therefore favouring T-cell apoptosis.

ARVs themselves might further compromise the action of 1,25[OH]_2_D, since some antiretroviral drugs (efavirenz, tenofovir and ritonavir) have been associated with changes in plasma levels of 1,25[OH]_2_D (Welz *et al.*, 2010; Dao *et al.*, 2011). In our cohort, amongst individuals with available dosage, we observed that 50% of the cases showing immunological failure used a NNTRI-containing regimen and 50% received a PI-containing treatment. Among the individuals with immunological success, approximately 42% used a NNTRI-containing therapy, against 58% who used PI.

We cannot fail to recognize our limitations. Firstly, the small sample number is directly linked to the individuals treatment adherence. Despite a large number of patients undergoing treatment, the majority had low ARVs adherence. Secondly, for some SNPs the number of genotyped individuals resulted different in relations to others due to the quality of the biological samples, implying technical difficulties in the genotyping process. A further limitation was due to the lack of VDR expression analysis caused by the impossibility to extract RNA from certain biological samples.

In conclusion, our results lead us to hypothesize (Figure 1) that genetic variations in VDR (rs11568820 – C allele and C/C genotype; CG allelic combination) might decrease VDR expression, influencing the binding of 1,25[OH]_2_D to VDR (possibly disrupting the 1,25[OH]_2_D action) and inducing apoptosis, consequently affecting CD4+ T-cells recovery. Our work reports for the first time the existence of a relationship between SNPs in the *VDR* gene and the immunological recovery in HIV-1 + individuals undergoing ARVs.

**Figure 1:**
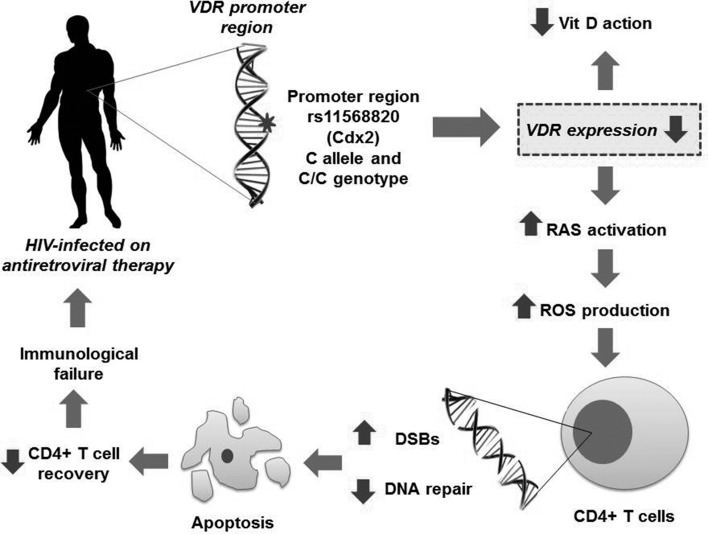
Hypothetical model of the VDR receptor involvement in HIV-infected individuals treated with antiretroviral therapy and showing immunological failure.

## Supplementary Material

The following online material is available for this article:

Table S1 Allelic combination of VDR functional SNPs.

Table S2 Adjusted clinical variables logistic regression model of VDR genotypes.

Table S3 Plasma levels of 25-hydroxy vitamin D.
