# Supplementary material for: VDR polymorphisms influence immunological response in HIV-1+ individuals undergoing antiretroviral therapy
Source: Genet Mol Biol. 2019 Jun 27;42(2):351–6. doi: 10.1590/1678-4685-GMB-2017-0289 (PMC6726152; doi:10.1590/1678-4685-GMB-2017-0289)
Supplement: Supplementary file 1 [file 1415-4757-GMB-1678-4685-GMB-2017-0289-20190513-suppl1.pdf]

Supplementary Material "*VDR* polymorphisms influence immunological response in HIV-1+ individuals undergoing antiretroviral therapy"

Table S1 - Allelic combination of VDR functional SNPs in HIV-1+ individuals under ARVs as to the virological and immunological response.

Combination		Virological			Immunological		
rs2228570	rs11568820	Success n(%)	Failure n(%)	Fisher Exact Test OR (95%CI), *p*-value	Success n(%)	Failure n(%)	Fisher Exact Test OR (95%CI), *p*-value
G	T	91 (38.9)	41 (38.0)	Reference	49 (43.0)	36 (34.6)	Reference
G	C	73 (31.2)	35 (32.4)	1.06 (0.59-1.90), 0.889	26 (22.8)	42 (40.4)	2.19 (1.09-4.45), 0.023*
A	C	56 (23.9)	23 (21.3)	0.91 (0.47-1.74), 0.877	29 (25.4)	22 (21.1)	1.03 (0.48-2.20), 1.000
A	T	14 (6.0)	9 (8.3)	1.42 (0.50-3.87), 0.474	10 (8.8)	4 (3.8)	0.55 (0.11-2.09), 0.391

OR = Odds ratios; 95%CI = 95% Confidence interval ; * = Significant *p*-value
