# Supplementary material for: VDR polymorphisms influence immunological response in HIV-1+ individuals undergoing antiretroviral therapy
Source: Genet Mol Biol. 2019 Jun 27;42(2):351–6. doi: 10.1590/1678-4685-GMB-2017-0289 (PMC6726152; doi:10.1590/1678-4685-GMB-2017-0289)
Supplement: Supplementary file 2 [file 1415-4757-GMB-1678-4685-GMB-2017-0289-20190513-suppl2.pdf]

Supplementary Material "*VDR* polymorphisms influence immunological response in HIV-1+ individuals undergoing antiretroviral therapy"

Table S2 - Adjusted clinical variables logistic regression model of *VDR* genotypes influence over immunological response

Variable	OR	95% CI	*p*-value
**rs2248098G/A**	0.66	0.21 - 2.06	0.48
**rs2248098G/G**	1.08	0.32 - 3.72	0.90
**rs11568820C/T**	2.15	0.78 - 5.95	0.14
**rs11568820C/C**	6.00	1.52 - 23.72	0.01*
**CD4+ T cells count (baseline)**	1.00	1.00 - 1.00	0.16
**Sex (male)**	0.54	0.21 - 1.38	0.20
**ART regimen**	2.04	0.83 - 4.99	0.12
**(Intercept)**	-	-	0.22

OR = Odds ratios; 95%CI = 95% Confidence interval; * = Significant *p*-value
