# Supplementary material for: VDR polymorphisms influence immunological response in HIV-1+ individuals undergoing antiretroviral therapy
Source: Genet Mol Biol. 2019 Jun 27;42(2):351–6. doi: 10.1590/1678-4685-GMB-2017-0289 (PMC6726152; doi:10.1590/1678-4685-GMB-2017-0289)
Supplement: Supplementary file 3 [file 1415-4757-GMB-1678-4685-GMB-2017-0289-20190513-suppl3.pdf]

Supplementary Material "*VDR* polymorphisms influence immunological response in HIV-1+ individuals undergoing antiretroviral therapy"

Table S3 - Plasma levels of 25-hydroxy vitamin D in a randomly selected sample subset of the individuals with immunological success and failure.

Vit D plasma level (ng/mL)	Immunological Success (n=19)	Immunological Failure (n=12)	Mann Whitney Test W, *p*-value
Median (IQR)	35.2 (28.1-41.1)	32.9 (31.4-41.0)	109, 0.86

n = Sample size; IQR = Interquartile range; W = Mann-Whitney test statistic
